# Why Did the Earwitnesses to the John F. Kennedy Assassination Not Agree About the Location of the Gunman?

**DOI:** 10.3389/fpsyg.2021.763432

**Published:** 2021-11-16

**Authors:** Dennis McFadden

**Affiliations:** Department of Psychology, Center for Perceptual Systems, University of Texas, Austin, TX, United States

**Keywords:** JFK assassination, US House Select Committee on Assassinations (HSCA), re-enactment of JFK assassination, acoustics of rifle bullets, sound localization, earwitnesses, shock wave, muzzle blast

## Abstract

Earwitnesses to the 1963 assassination of President John F. Kennedy (JFK) did not agree about the location of the gunman even though their judgments about the number and timing of the gunshots were reasonably consistent. Even earwitnesses at the same general location disagreed. An examination of the acoustics of supersonic bullets and the characteristics of human sound localization help explain the general disagreement about the origin of the gunshots. The key fact is that a shock wave produced by the supersonic bullet arrived prior to the muzzle blast for many earwitnesses, and the shock wave provides erroneous information about the origin of the gunshot. During the government's official re-enactment of the JFK assassination in 1978, expert observers were highly accurate in localizing the origin of gunshots taken from either of two locations, but their supplementary observations help explain the absence of a consensus among the earwitnesses to the assassination itself.

## Introduction

President John F. Kennedy (JFK) was assassinated in Dallas, Texas, on 22 November 1963. Within days, the new president, Lyndon B. Johnson, appointed a blue-ribbon panel of seven legislators and statesmen to investigate the assassination. The commission was headed by the chief justice of the US Supreme Court, Earl Warren, and consisted of about 400 staff and a budget of about $10 million. The commission held public hearings and officially interviewed over 500 people. About 10 months after the assassination, the commission published a report of nearly 900 pages plus 26 volumes of interviews, depositions, and exhibits, all of which came to be called the Warren Report (Warren Commission, 1964). This was arguably the most thoroughly investigated murder in the history of the world.

The primary conclusions of the Warren Commission were: there was only one assassin, Lee H. Oswald, who shot from a corner window on the sixth floor of the Texas School Book Depository (TSBD) building, a location behind the president. Oswald fired three shots using the Mannlicher-Carcano rifle found in the TSBD, one shot missed and two struck the president. The second bullet struck the president in the upper back, exited from the front of his throat, and then struck Texas Governor John Connally, who was seated in front of, below, and inboard of the president (the single-bullet account). The third shot struck the president in the head, killing him within minutes. The commission noted that an 8-mm film taken by Abraham Zapruder greatly aided their investigation.

Even before the Warren Report was released, questions emerged about various people and events surrounding the assassination, and these led to a number of conspiracy theories about aspects of the JFK assassination. The Warren Commission emphasized that they found no evidence of any conspiracy between Oswald, organized crime, Cuba, the FBI, the CIA, the military-industrial complex, Jack Ruby (Oswald's assassin), or any other entities. Even so, conspiracy theories continued to thrive. In April 1968, Martin Luther King, Jr. (MLK) was assassinated in Memphis, Tennessee, and again there was widespread disbelief about several details of the official account of that event.

The persistence of public disbelief in the official accounts of the JFK and MLK assassinations led the US House of Representatives to create a House Select Committee on Assassinations (HSCA) in 1976 (Bugliosi, 2007a, p. 370+). The hope was that scientific advances made since the Warren Report and the MLK investigation would allow additional information to emerge that could help resolve some of the prevalent questions. The HSCA employed about 250 people, spent about $5.8 million, and issued its report in 1979. Their activities included a partial re-enactment of the assassination in Dealey Plaza in August 1978 using live ammunition. A primary motivation for the partial re-enactment was a dictabelt recording inadvertently transmitted from a motorcycle of the Dallas Police Department on the day of the assassination that might have contained the sounds of the gunshots (explained in McFadden, 2021).

The HSCA report (House Select Committee on Assassinations, 1979) received world-wide attention because at the last minute it was rewritten around a conclusion of there having been four gunshots, not the three mentioned in the Warren Report (Bugliosi, 2007a, p. 380; Bugliosi, 2007b, p. 153+). For various reasons, four shots meant two shooters, exactly what many conspiracy theorists had been arguing for years. As explained in Supplementary Material (McFadden, 2021), that conclusion was shown to be unquestionably erroneous within months of the publication of the HSCA report, but unfortunately the bell could not be unrung, and the HSCA report has contributed to the widespread belief that the Warren Report was wrong.

The HSCA's partial re-enactment of the assassination was primarily concerned with the physical acoustics of gunshots in Dealey Plaza (Barger et al., 1979), but also present was a psychoacoustics team, consisting of David M. Green, Frederic L. Wightman, and your author. The activities and the findings of the psychoacoustics team are primary components of this article (see below).

The assassination of President John Kennedy was unquestionably one of the major historical events of the 20th century, and unfortunately there are many erroneous claims and theories about the event scattered throughout the public record. Among the more serious errors is the conclusion by the HSCA that there were four gunshots taken at the President, not three. There are multiple goals for this article: to discuss the acoustics of rifle bullets and the psychoacoustics of localizing the source of rifle bullets in highly reverberant locations in order to help explain why earwitnesses to the JFK assassination were so uncertain about the location of the gunman; to retell the story of the HSCA partial re-enactment of the JFK assassination and to explain the mistake made by the physical acousticians; to provide some personal perspective about the partial re-enactment; and to provide an example of applying knowledge obtained in laboratory studies to real-world situations, in this case an important historical event. Some of the content of this article repeats publicly available information that is not generally known to recent generations of acousticians, psychoacousticians, or the citizenry. Specifically, the report from the psychoacoustics team to the HSCA (Green, 1978, p. 111–130; Green, 1979) contained a section describing the difficulties humans have localizing the origin of a supersonic rifle bullet in a highly reverberant space, along with the observations made by the psychoacoustics team during the re-enactment, discussions that are repeated and elaborated here and in Supplementary Material (McFadden, 2021). My belief is that these stories deserve retelling so that citizens are more knowledgeable about the facts of their history.

## Earwitness Reports

**Figure 1:**
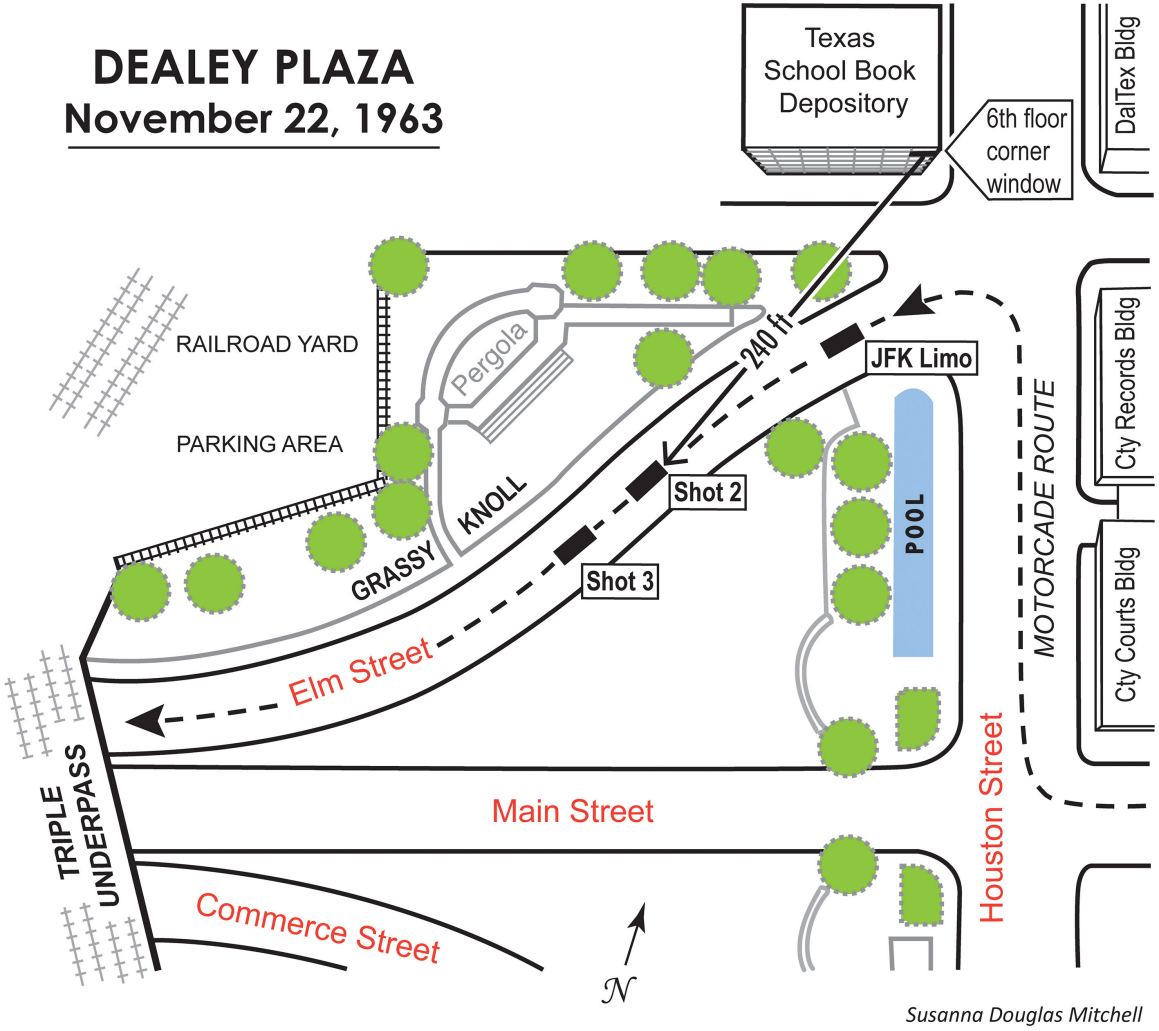
Diagram of Dealey Plaza, showing the path of the motorcade (dashed line); the location of the assassin on the 6th floor of the TSBD; three locations of the presidential limousine corresponding roughly to its locations when the three shots were fired from the TSBD; the location of the pergola where A. Zapruder stood while filming the assassination; and other relevant details. Figure adapted from Green (1979) by Susanna Douglas Mitchell.

Figure 1 illustrates the scene of the assassination, Dealey Plaza. The president's motorcade began at the Dallas airport (Love Field), and consisted of over a dozen vehicles carrying local, state, and national dignitaries, the press, and Secret Service agents. The JFK limousine was second in line, and it carried the President and his wife, Texas Governor Connally and his wife, and two Secret Service agents. The motorcade left downtown Dallas on Main Street, turned right on Houston Street for one block, and then turned left onto Elm Street in front of the TSBD. The assassin's first shot came soon after the turn onto Elm Street, at ~12:30 p.m. Upon hearing the shots, the Secret Service agents accelerated, and the presidential limousine took Stemmons freeway to Parkland Hospital where the President was pronounced dead about 1:00 p.m. Vice-President Johnson was sworn in as president just prior to takeoff on Air Force 1, which then returned the presidential party to Washington, D.C.

Estimates are that somewhere between 500 and 700 people were in Dealey Plaza at the time of the assassination. Many were employed nearby and came out during their lunch hour to watch the motorcade pass by. Most of the spectators were arrayed along Houston Street, with fewer on the sidewalk and grass on either side of Elm Street.

Soon after the assassination, subsets of these witnesses were interviewed by various investigators. This was not a well-planned research study, so the interviews generally lacked consistency. However, they do provide the best subjective evidence available about the assassination. One of the better compilations of these interviews comes from the work of Thompson (1967, p. 25). He summarized the responses of 190 witnesses who either testified to the Warren Commission or spoke with a law-enforcement officer whose report reached the Commission's files. Fortunately, these interviews contained information about the general location of each witness, which is important because of the acoustic complexity of Dealey Plaza. Unfortunately, the interviews occurred at different times after the assassination, meaning that the earwitnesses had been exposed to differing numbers of newspaper and television reports about the investigation to date. Thus, their accounts may have been different from what they would have been if obtained immediately after the event. Also, all relevant questions about the gunshots apparently were not asked of every earwitness, or at least all the responses were not noted. Accordingly, caution is required not to over-interpret the results. It is important to understand that, unlike what would happen today, no commercial or personal audio equipment was operating in Dealey Plaza at the time of the assassination (and the Zapruder film was silent). All we have are earwitness reports plus the physical evidence.

The obvious needs to be said here, and acknowledged as important. Each of the earwitnesses to the JFK assassination was startled, surprised, confused, disbelieving, excited, and fearful, to varying degrees. Further, they brought to the event different previous experiences with gunshots. Finally, memories are known to change with time. All of these factors unquestionably contributed to the perceptions and memories reported, and that needs to be recognized when evaluating the verbal descriptions of the auditory (and visual) events provided by the earwitnesses. There should be no surprise that the recollections about details during those critical few seconds differed across individuals. As an extreme example of the differences across individual earwitnesses, Thompson reported that the descriptions of the time interval between the first and last shot varied from seconds to minutes! By contrast, there was good agreement about the number of shots heard, with 79% of the 172 people who answered that question saying 3 shots. Similarly, about 61% of the 65 people answering agreed that the final two shots were closer in time than were the first two shots (Thompson, 1967, p. 25, Appendix).

Although the earwitnesses were in reasonable agreement about the number and spacing of the gunshots, there was less consistency in their comments about arguably *the* most crucial question about the assassination: the origin of the gunshots. Only 64 of Thompson's 190 earwitnesses gave any opinion at all on that issue. That is, fully *two-thirds* of the earwitnesses apparently were too uncertain of the source of the gunshots to offer a location for the gunman (or maybe the answer was so obvious that it did not require comment?). This was a truly peculiar fact that deserved serious attention from the HSCA psychoacoustics team. What factors might have led so few earwitnesses to express an opinion about the origin of the gunshots when most of them did have opinions about the number and spacing of the shots?

By the way, of Thompson's earwitnesses who did mention a location, about 52% identified the grassy knoll or triple underpass region and about 39% mentioned the TSBD building. When evaluating various conspiracy theories, it is important to know that, by one count, *only four* earwitnesses mentioned more than one location for gunmen (Green, 1979, p. 10; Bugliosi, 2007b, p. 174).

Having acknowledged the potential contribution of rapidly aroused emotion to the earwitness reports, are there other factors that could have contributed to the general uncertainty and disagreement the earwitnesses exhibited about the origin of the gunshots? Yes, but first some background.

## Localizing Sound Sources

In less emotion-filled settings, humans are extraordinarily skilled at localizing the origins of sounds in three-dimensional space. As readers of this journal are aware, humans use three basic cues for sound localization. The direction of the source along the left/right axis is determined by the differences at the two ears in both the time of arrival of the sound and the sound-pressure level (SPL) of the sound, with spectral cues indicating source location in the vertical and front/back directions (Wightman and Kistler, 1992, 1999). The first cue (timing) exists because the speed of sound in air is relatively slow compared to the distance between our two ears; it takes more time for a sound to reach the further ear than the nearer ear. These time differences can be as large as 600–800 μsec, depending upon the size of the observer's head, but most humans are sensitive to interaural time differences as small as 10 μsec. The second cue (SPL) exists because the head throws a “shadow” when its width becomes large compared to the wavelength of the incident sound. At high frequencies, the magnitude of the interaural level difference can be tens of decibels, but most humans are sensitive to interaural differences of <1 dB. In the everyday world, interaural time and level cues typically operate in unison; the nearer ear typically also receives the stronger sound. The third cue of spectral differences originates in the acoustics of the pinnae; these cues are specific to the individual observer, and are essential for resolving confusions of front and rear source locations (Wightman and Kistler, 1999).

## The Acoustics of Gunshots

Considerable insight into the earwitnesses' uncertainty about the location of the gunman is gained by examining the acoustics of rifle bullets. Gunshots are not like the stimuli commonly used in laboratories to measure human abilities to localize sounds. The bullets commonly fired from rifles travel at supersonic speed, meaning that there are two sources of sound associated with every rifle shot. First there is the *muzzle blast* that originates from the explosion inside the barrel and the exit of the bullet from the barrel (Beck et al., 2011). This sound behaves in a familiar manner. It propagates away from the rifle spherically, traveling at the speed of sound in air (~1,125 feet per second or 343 meters per second), while losing about 6 dB of level for each doubling of the distance from the source.

The second source of sound for a supersonic bullet is a *shock wave* that is generated as the bullet moves through the air (Sapozhnikov et al., 2019). At the front of the bullet is a substantial overpressure produced because the surrounding air cannot move fast enough to “get out of the way.” At the rear of the bullet is a substantial underpressure because the rapid flight of the bullet leaves a partial vacuum that takes time for the surrounding air to equalize. The overpressure at the front of the bullet and the underpressure at the rear of the bullet produce a rapid N-shaped pressure change that propagates away from the bullet. As we will see, the existence of these N waves has the potential to explain much of the uncertainty the earwitnesses had about the origin of the gunshots during the JFK assassination, so it is worthwhile to provide more details about N waves (also see Green, 1979; Sapozhnikov et al., 2019; McFadden, 2021).

For small objects like bullets, the time between the peak overpressure and the peak underpressure is so small that the two disturbances can be conceptualized as a single impulse. Once that N wave reaches a human observer, the perceptual experience commonly is described as a boom, snap, or crack. For large objects like supersonic aircraft, the peak overpressure and the peak underpressure of the N wave both can produce impulses, milliseconds apart (the double boom familiar to people living near military airbases). In general, the N waves produced by large objects have larger peak overpressures and larger peak underpressures than N waves from small objects.

**Figure 2:**
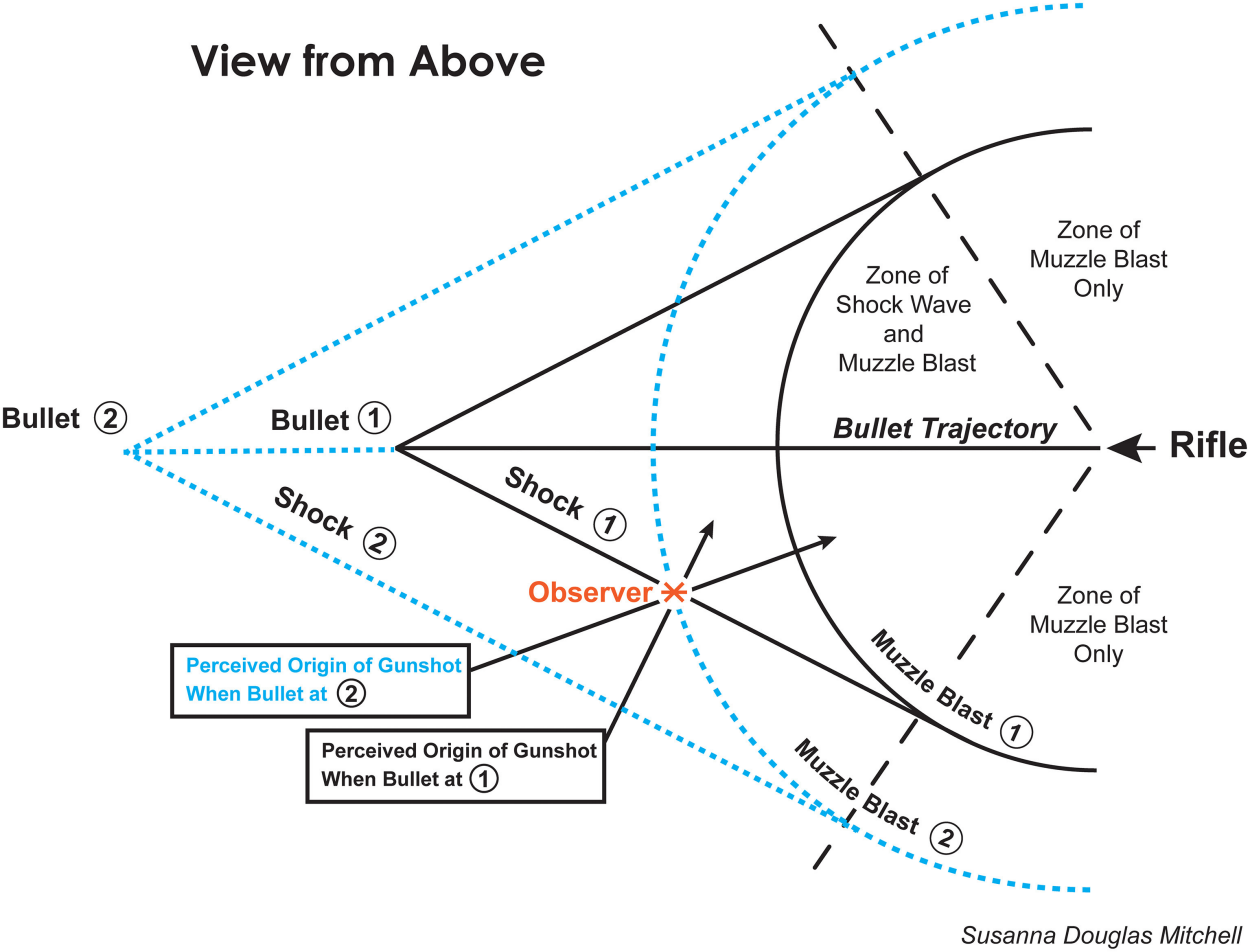
A diagram of the muzzle blast and the shock wave associated with a gunshot from a rifle firing supersonic bullets. The rifle is located at the right of the figure and the bullet proceeds toward the left. The curved lines in the figure illustrate the muzzle blast propagating spherically away from the rifle, and the cone-shapes illustrate the path traversed by the shock wave propagating away from the path of the bullet. Because the bullet travels faster than the speed of sound, the sound produced by the shock wave arrives prior to the sound produced by the muzzle blast at most locations in front of the gunman. When the bullet reaches position 1, an ideal observer located at the red asterisk would hear the sound generated by the shock wave and would localize the source as the path of the bullet. This judgment is correct based on the stimulus, but incorrect about the origin of the bullet. When the bullet reaches position 2, an ideal observer located at the red asterisk now hears the muzzle blast and localizes that source as the location of the gunman. Of course, humans are not ideal at separating the two sources of sound, so the potential for confusion is high (The duration of the N wave increases moving from left to right along the edges of a cone; see text). Figure adapted from Green (1979) by Susanna Douglas Mitchell.

Some additional details: (1) Technically, both the positive and negative peaks of the N wave are called shock waves, but for the purposes of this article “shock wave” typically will be used to denote the initial overpressure of the N wave. (2) The shock wave associated with the overpressure at the front of the bullet travels slightly faster than the speed of sound in the surrounding air, and the shock wave associated with the underpressure at the rear of the bullet travels slightly slower. Thus, the duration of the N-shaped wave increases with distance traveled from the source; the shock wave associated with the overpressure increasingly outraces the shock wave from the underpressure. (3) Unlike the familiar spherical pattern of propagation for the muzzle blast, the N wave propagates away from the supersonic bullet (or airplane) in a pattern having the shape of a cone (the Mach cone), with the tip of the cone at the front of the bullet. (4) In general, an N wave that spreads with the shape of a Mach cone loses about 4.5 dB of level for each doubling of distance from the source, as compared to the familiar −6 dB for the spherically spreading muzzle blast. This means that the *relative* strength of the N wave compared with the muzzle blast becomes greater with increasing distance from the rifle. Figure 2 illustrates both the muzzle blast (curved lines) and the shock wave (cone shapes) from a supersonic bullet at two instants in time as the bullet travels from right to left across the figure (For an animation of an N wave, see https://en.wikipedia.org/wiki/Sonic_boom#/media/File:Sonicboom_animation.gif).

One important point to note from Figure 2 (adapted from Green, 1979) is that the curved and conical wavefronts provide localization cues for different points of origin of the sounds. The curved, muzzle-blast waves did originate at the location of the gunman, and they carry values of interaural time difference and interaural level difference appropriate for that location. By contrast, the conical patterns originated from *the path of the bullet*, and they carry values of interaural time difference and interaural level difference appropriate for the location of that path, not the location of the rifle (see Figure 2).

The second important point to note from Figure 2 is that, at all locations more than a few meters in front of the rifle, the sound from the shock wave arrives *before* the sound from the muzzle blast. Also, the time difference between the two increases with increasing distance from the source (the ratio of the time differences is constant). At a distance of about 240 feet (about 73 meters; the approximate distance between the TSBD and the president at the time of the shot that hit him in the neck), the sound of the shock wave from a bullet from a Mannlicher-Carcano rifle will precede the muzzle blast by about 100 ms. The N wave and muzzle blast for a Mannlicher-Carcano rifle are shown in McFadden (2021) (Supplementary Figure 1).

## Acoustical Reasons for Confusion

To repeat, the muzzle blast, like all other everyday sounds, gives rise to values of interaural time and level difference and spectral cues that an observer can use to accurately localize the source of that sound. However, the location cues extracted from the earlier-arriving shock wave are informative only about *the path of the bullet*, not the origin of the bullet. Thus, observers presuming that the interaural information extracted from the first-arriving sound at their location was informative about the location of the rifle would be led to compute an erroneous location for the gunman. In the case of Dealey Plaza, that computed location could even be logically absurd.

Consider an observer standing on the sidewalk along Elm Street between the TSBD and the grassy knoll, in front of the pergola and facing Elm Street (see Figures 1, 2). The location cues extracted only from the first-arriving shock wave would lead to a conclusion that the rifle was slightly overhead, somewhere in the sky above the buildings in the distance to the southeast. The lifetime of knowledge a human observer brings to such a situation runs contrary to such an impossible conclusion, and confusion would be the result. Also, soon after the shock wave, the muzzle blast would arrive from the left carrying quite contradictory information about the source of the sound. The potential for confusion was high [Abraham Zapruder was filming from in front of the pergola, and at one time he said that the shots seemed to come from behind him (which the TSBD was not), although later he said he could not be certain about their origin (Bugliosi, 2007b, p. 169).].

In an acoustic environment like Dealey Plaza, there also are reflections of both the muzzle blast and shock wave off the various hard surfaces in the vicinity (Barger et al., 1979, Figure 2; Beck et al., 2011). These reflections reach each observer in rapid succession, with a temporal pattern that will depend upon the location of the observer. Note that the location cues associated with each reflection also are not informative about the initial source of the sounds: the rifle. Rather, they are informative only about the *origin of that particular reflection*. The shock wave and muzzle blast are spectrally and temporally different sounds; by contrast, reflections are repetitions of the same sound after it has bounced off nearby surfaces.

The time difference between the shock wave and the muzzle blast, and the timing of the various reflections depend upon the distance and location of the observer in relation to the gunman, meaning that the auditory experience of each observer in Dealey Plaza was different. With each gunshot, every observer was exposed to a complex pattern of successive sounds, all carrying location information, but only one of those incident wavefronts, the direct path from the muzzle blast, carried *correct* information about the initial source of all those sounds: the rifle.

This analysis of the acoustics of the events in Dealey Plaza helps us understand why the earwitnesses to the JFK assassination were uncertain about the origin of the gunshots that day.

## Psychoacoustical Reasons for Confusion

Auditory science knows much about how humans react to presentations of multiple sounds in rapid succession. One relevant phenomenon is known as the *precedence effect*. This is a binaural effect that operates over the course of *microseconds*. When two sounds from different locations in space occur in rapid succession, the location cues extracted from the first sound dominate the perception of the location of the fused sounds (Wallach et al., 1949; Litovsky et al., 1999). Also, architectural acousticians know that an auditorium having strong reflections that occur more than about 30 ms after the arrival of the direct sound will be perceived as “hollow,” and with increasing time delays, echoes will be reported.

Another phenomenon known to every architectural acoustician operates over the course of tens of *milliseconds*, and does not depend upon binaural cues (McFadden, 1973). Consider a public-address system in a large room with a soloist performing before a microphone at the front of the room. If a loudspeaker at the rear of the room were energized immediately with the sound collected by the microphone, listeners at the rear of the room would receive the sound from that loudspeaker before receiving the direct sound from the soloist, and they would perceive the sound as originating from the loudspeaker, not from the front of the room. To eliminate this unnatural experience, public-address systems introduce a time delay between the sound picked up by the microphone and the electrical waveform delivered to the distant loudspeakers. When the delay is a few tens of milliseconds, the listeners close to the distant loudspeakers perceive the sound as originating from the (distant) soloist rather than from the loudspeaker located nearby. This illusion exists even when the direct sound is tens of decibels weaker than the amplified sound from the loudspeaker; also, this illusion exists for monaural listeners as well as binaural listeners. This effect could lead earwitnesses to emphasize the shock wave over the muzzle blast and thus reach an erroneous conclusion about the origin of the gunshots.

Another factor relevant to understanding the varied perceptions of the earwitnesses about the origin of the gunman is “*front/back*” *confusions*. The geometry of sound localization using interaural time differences is such that there are inherent ambiguities about the source of the sound. For every value of interaural time difference, there always are a large number of locations in three-dimensional space from which the sound could have originated. For example (to the extent that the head is a sphere), one sound source located at 30 degrees to the right of the listener and another located at 150 degrees to the right both give rise to the same value of interaural time difference. Indeed, the locus of all locations possibly giving rise to the value of time difference in this example is described by a cone having its apex located at the right ear canal and its base off to the right of the head. These loci often are called cones of confusion. In everyday environments almost all relevant sounds originate from sources on the ground (essentially at “ear level”), so most of that cone can be ignored, and people typically do. However, the front/back ambiguities still exist.

When a sound is ongoing, small head movements can resolve the front/back confusion (Wightman and Kistler, 1999), but rifles make impulsive sounds. It is believable that, at the time of the JFK assassination, some of the earwitnesses who localized the path of the bullet using the shock-wave information and then realized the gunman could not be suspended in the sky or in some other impossible location simply concluded instead that the sound came from behind them, a front/back reversal based on the shock wave (Green, 1979). These would not have been conscious decisions; each earwitness had a lifetime of subconscious experience with cones of confusion. Garinther (cited in Green, 1979, p. 5) observed front/back reversals about 25% of the time in listeners trying to localize gunshots whose muzzle blasts had been attenuated. Yost and Pastore (2019) reported similar percentages using much longer stimuli. As noted, A. Zapruder initially reported that the gunshots seemed to originate from behind him (Bugliosi, 2007b, p. 169).

All of these various psychoacoustical effects reveal that a first-arriving sound is given more emphasis by the human auditory brain than are any later-arriving sounds. This makes sense from the perspective of evolution because in most real-world settings the direct sound arrives prior to its reflections, which carry erroneous localization information. However, this characteristic of human auditory perception can lead to errors of localization in certain unusual situations, and gunshots in reverberant environments are one of those situations.

Taken together, then, a consideration of the physical acoustics of rifle bullets and the psychoacoustics of sound localization helps explain the uncertainties and inconsistencies among the earwitnesses to the JFK assassination (Green, 1979).

## The Partial Re-Enactment

Two teams of investigators were involved in the partial re-enactment of the JFK assassination in Dealey Plaza: physical acousticians and psychoacousticians. The physical acousticians were there to make high-quality recordings using an array of microphones while gunshots were taken at sandbags located at positions known to be relevant. The goal was to determine whether any of the sounds recorded during the re-enactment could be matched to any of the impulse patterns on the police dictabelt, thereby demonstrating that the motorcycle with the stuck Transmit button was in Dealey Plaza at the time of the assassination. If it was, there was a possibility that some additional knowledge could be obtained about the number, origin, and path of the bullets fired during the assassination.

The psychoacousticians were at the re-enactment because the staff of the HSCA believed that having some “expert listeners” present during the re-enactment might provide explanations for some of the contradictory reports of the earwitnesses present at the assassination. As noted, some earwitnesses were adamant that all the gunshots came from the TSBD, some were convinced that shots came from the vicinity of the grassy knoll, and some gave other reports. These discrepancies contributed to the breadth and persistence of the conspiracy theories that had emerged since the assassination. The hope was that some of these discrepancies would be better understood after the re-enactment. The activities and findings of the psychoacoustics team were described to the HSCA by Green (1978, 1979); additional details are in Supplementary Material (McFadden, 2021).

During the partial re-enactment in 1978, marksmen shot either from the sixth-floor window of the TSBD or from behind a fence on the so-called grassy knoll commonly viewed as a possible location for a second gunman (see Figure 1). There were four sandbag targets, and several sequences of shots were made as the 12-microphone array was positioned in three different locations. The positions of the microphones are shown in McFadden (2021) (Supplementary Figure 2). Mannlicher-Carcano rifles like the one Oswald left in the TSBD were used along with a pistol fired only from behind the fence on the grassy knoll. During those sequences of shots, the psychoacousticians were located together or separately at different positions in Dealey Plaza. Our tasks were to indicate the perceived origin of each shot (forced choice: TSBD or grassy knoll) and to note additional aspects of each perceptual experience (the nature, number, direction, and duration of the echoes, etc.). The primary observers were Drs. Wightman (FLW) and McFadden (DM) (Dr. Green has a unilateral hearing loss.). Although observations were made from multiple locations, this was not a carefully controlled, counter-balanced study. As is true for most applied-science projects, we had to get a feel for the situation during the early gunshots, and our experiences led to some last-minute changes in the initial plan.

## Observations During the Partial Re-Enactment

The plan was for three sequences of 12 gunshots each (see Supplementary Table 1 in McFadden, 2021), but in the event, repeat shots were taken when the physical acousticians needed them. Observers FLW and DM were ignorant of the planned sequences, and we did not know the exact moment of each shot, but we were told when a shot was about to be taken. The individual shots were separated by differing intervals, but the average was 1–2 min. Including the repeats required by the physical acousticians, we observed 57 gunshots. Some locations gave rise to greater uncertainty about the origin of the shots than others, but overall, the two observers did quite well at localizing the source of the gunshots (see McFadden, 2021; Supplementary Table 2). Averaged over the sequences and locations, observer FLW was 86% correct at identifying the origin of the shots and DM was 93% correct. For most of the test shots we were not located together (Green, 1979, p. 14), and there was no comparison of responses and perceptions until the end of the re-enactment. To be sure, we faced a simpler task than the earwitnesses because we had far less uncertainty about the possible source of the shots, and there was no factor of surprise. The shot-by-shot field notes for both observers are in Appendix A in Green (1979).

One primary observation was that the gunshots were extremely loud from all our observer positions. They were so loud that we were frankly mystified by the reports from several earwitnesses of initially having thought they heard firecrackers. Those people must have had experience with firecrackers far larger than any the psychoacoustics team was allowed to play with. Admittedly, Dealey Plaza was much quieter during the re-enactment than on the day of the assassination, so there was less masking. HSCA staff intentionally did have three idling motorcycles to add some masking noise, but they contributed little.

Almost all of the gunshots during the re-enactment gave rise to sounds whose origins were diffuse, not narrowly focused or precise. No matter what our observer positions or the marksmen's target, our perceptions were of general *areas* for the origin of the gunshots, never anything as precise as the corner window on the 6th floor of the TSBD or the corner of the fence behind the grassy knoll. Indeed, from some observer positions the origin might appear off to the east of the TSBD or from the underpass down Elm Street, but our forced-choice decisions in those situations were TSBD and grassy knoll, respectively. [Some earwitnesses to the assassination also gave vague responses that did not specifically mention the TSBD or the grassy knoll, but for expediency Thompson (1967) encoded them as TSBD or knoll].

Although observers FLW and DM did quite well at identifying the source of the gunshots during the re-enactment, we also were impressed by the complexity of the acoustic perceptions aroused by the different shots. Multiple reflections from multiple directions often could be heard. In accord with expectation, the re-enactment rifle shots taken with the muzzle inside the window frame typically were noticeably less loud than those taken with the muzzle outside the window. Because this manipulation would affect the muzzle blast but have no effect on the shock wave, it reveals that much of the loudness percept was attributable to the later-arriving muzzle blast. Not surprisingly, at some observer positions, the perceptions changed noticeably when the marksmen changed targets, thereby changing the path of the bullet.

Perhaps most importantly, both primary observers were overwhelmed by those rifle shots originating from the grassy knoll. Those were *very* loud and unambiguous. We are convinced that had any rifle shots actually originated from the knoll area on the day of the assassination, the earwitnesses from that vicinity would have shown high confidence and high agreement about that fact. The fence on the grassy knoll was only a few meters to the right of the amateur photographer, A. Zapruder. Had a rifle shot actually originated from the grassy knoll, his startle response might well have knocked him sideways, off his perch on the pergola.

The pistol shots from the grassy knoll during the re-enactment were noticeably different from the rifle shots, being much less loud and much more narrowly focused than the rifle shots. This was evident to both observers from every observer position. We believe that earwitnesses would have been far more accurate and consistent in their reports had any subsonic pistol shots been fired at the motorcade.

In McFadden (2021) are some additional details about the methods of the psychoacoustics team, some detailed analyses of the earwitness reports from 1963 (Supplementary Table 3), and some comparisons between the perceptions of the psychoacoustics team and the earwitnesses.

## Summary

Localizing the origin of a supersonic gunshot is not easy under optimal conditions. On the day of the JFK assassination, the earwitnesses present were startled, surprised, confused, disbelieving, excited, and likely scared, so there is little wonder that their perceptions were inconsistent, and with the passage of time, fluid. Once the confusing acoustics of supersonic bullets and the vagaries of human sound localization are taken into account, the widespread uncertainty amongst the earwitnesses to the assassination becomes more understandable. The key point is that for many earwitnesses, the N wave arrived first, and it carried erroneous information about the location of the gunman. It is truly unfortunate that the physical acoustics measured during the partial re-enactment of the JFK assassination led to an official government report that was incorrect, but in the end, history received another sterling example of self-correction in science. Also, I believe that the psychoacoustics work done during the re-enactment was helpful in clarifying the earwitness testimony from the day of the assassination. Nominally “expert” listeners knowing that the gunshots could originate from only one of two locations were quite accurate in identifying the source. Furthermore, we identified some of the misperceptions that could arise for observers at various locations in Dealey Plaza. For those of us on the psychoacoustics team, the partial re-enactment was a welcome opportunity to apply our lab-based knowledge to a real-world problem of enduring international interest.

## Author's Note

The account presented here is the author's retelling of the report by Green (1978, 1979) plus historical information from before and after the HSCA partial re-enactment; no current entity of the US Government contributed to this material.

## Author Contributions

The author confirms being the sole contributor of this work and has approved it for publication.

## Funding

The author did receive *per diem* from the US government for the time spent at the 1978 partial re-enactment.

## Conflict of Interest

The author declares that the research was conducted in the absence of any commercial or financial relationships that could be construed as a potential conflict of interest.

## Publisher's Note

All claims expressed in this article are solely those of the authors and do not necessarily represent those of their affiliated organizations, or those of the publisher, the editors and the reviewers. Any product that may be evaluated in this article, or claim that may be made by its manufacturer, is not guaranteed or endorsed by the publisher.
